# A Narrative Review of Vitamin A Supplementation in Preterm and Term Infants

**DOI:** 10.7759/cureus.30242

**Published:** 2022-10-12

**Authors:** Abhishek Kumar, Ashish Anjankar

**Affiliations:** 1 Department of Biochemistry, Jawaharlal Nehru Medical College, Datta Meghe Institute of Medical Sciences, Wardha, IND

**Keywords:** maturation, deficiency, supplementation, infants, vitamin a

## Abstract

Vitamin A deficiency is an epidemiologically significant concern in all age groups, especially in preterm and term infants. Its deficiency causes various developmental malformations. Vitamin A supplementation has been a practiced alternative for many decades, but its effectiveness is debatable in the medical system. The bioavailability of beta-carotenes varies greatly and ranges from 2% to 30%, depending on how it is present in the plant's cellular composition. Vitamin A has a bioavailability of up to 75%. The bioavailability of beta-carotenes is positively impacted by several activities but mainly by mechanical ones that allow cellular interaction. These include enough chewing, mincing, and pureeing. The bioavailability of beta-carotene can be increased by moderate cooking and combining high-quality lipids. The WHO recommends waiting for a minimum of one month between vitamin A dosages. Six months is the maximum amount of time between dosages. For instance, giving the optimum dosage to a child who has not had vitamin A in two months is preferable to skipping the dose and making the child wait eight months (i.e., two months plus six months) before receiving the following amount. There were no discernible variations in the occurrence of momentarily increased aspartate aminotransferase (AST), alanine transaminase (ALT), or alkaline phosphatase between the leading group and the trace group. However, patients in the top group experienced high blood triacylglycerol levels more frequently than those in the trace group, suggesting that hypertriacylglycerolemia may be a side effect of vitamin A administration. It is imperative to memo that neonatal vitamin A supplementation negatively affects subsequent diphtheria-pertussis-tetanus (DPT) vaccination in females. Since many children are delayed in obtaining their initial DPT series, several nations prescribe DPT boosters, so older children may be affected if vitamin A supplementation negatively interacts with DPT in such children.

## Introduction and background

In poor nations, predominantly those in the African subcontinent Asia-Pacific region, vitamin A deficiency is an unembellished well-being concern [1]. Infants have been fed cod liver oil or particular essential fats to avoid childhood bone deformities, and fish liver oils also provide a significant amount of vitamin A. Since the majority of low-fat milk sold in the United States is reinvigorated with vitamin A, even the consumption of low-fat milk, which is not recommended in the initial year of its lifetime, now calls for vitamin A fortification [2]. Nearly two billion people worldwide, according to estimates from the World Health Organization (WHO), are suspected of having vitamin A deficiency in the coming future. Approximately 125 million preschoolers suffer from vitamin A insufficiency [3]. One of several nutrients most thoroughly investigated in terms of immune system function is vitamin A. Even before the structure of vitamin A was determined in 1931, the first findings that showed a connection between vitamin A and immunity were discovered [4]. Vitamin A is essential for the defense mechanism of the body, vision, and general growth and progress of newborns and children to work at their best [5]. Vitamin A plays a pivotal role in immune protection and the development and differentiation of epithelial tissues [6]. Indications of inadequate amount of vitamin A in the body have been utilized as an initial signal in such emergencies because a drop in the nutritional value of food, leading to deficiencies in vitamins, is an early reaction to crises that impact diet goods and causes family food instabilities [3]. The impact of giving children between the ages of six months and five years old high-dose vitamin A supplements on overall mortality was examined in several randomized studies conducted in the late 1980s and early 1990s. In 1993, the WHO advised biennial vitamin A supplements for all children between six months and five years after a meta-analysis found a correlation between vitamin A supplementation and a 23% decrease in child mortality [7].

There are little data that can be used to evaluate the effects of supplement usage on child nutrition, even though infancy is a crucial time for growth and development, and that nutrient shortage, excesses, or imbalances may have significant long-term impacts on health [2]. Additional vitamin A supplementation may lessen prematurity-related problems such as retinopathy, intraventricular hemorrhaged necrotizing enterocolitis, and respiratory infections [8]. Too much vitamin A can potentially be dangerous since it can increase intracranial pressure, cause damage to or lesions of the skin and mucous membranes, and cause vomiting [8]. Increased morbidity and mortality from the respiratory and diarrheal consequences of measles are linked to mild to severe vitamin A deficiency. These issues result in a greater need for vitamin A and a decreased consumption due to reduced appetite [9]. Animal and in vitro research have substantially benefitted the study of vitamin A immunology. An extensive collection of familiarity with the cell processes by which vitamin A and its products regulate immune function at different levels has been produced through these investigations [4]. One IU of vitamin A corresponds to 0.3 µg of retinol which is equivalent to 3.33 µg. Supplemental beta-carotene has half the potency of retinol. According to the beta-carotene record, 1 µg of beta-carotene is equivalent to 1.66 IU of vitamin A (1 IU = 0.6 µg) and 0.5 µg of retinol (286 µg/L of retinol is 1 µM/L [10]). A biochemical vitamin A insufficiency is indicated by a serum retinol concentration of less than 0.7 µM/L (200 µg/L), while a moderate deficiency is characterized by a value of 0.7 to 1.05 µM/L (200 to 300 µg/L). Milk with below 1.05 µM/L (around 300 µg/L) has a low vitamin A concentration [10]. Accepted biomarkers may be affected by the subject's hydration, age, gender, kidney function, and other factors like the time of year or weather times of yearly food shortages [11]. People on poor-quality diets rarely have just one deficiency; they often have multiple micronutrient deficiencies [6].

## Review

Epidemiology

The population groups in the utmost danger of vitamin A deficit comprise offspring under five, expecting women, and nursing mothers. Low serum retinol concentrations affect children aged six to 59 months and are classified into three categories: mild, moderate, and severe. Vitamin A prevalence of 2% to 9% is considered a mild problem, 10% to 19% is a reasonable issue, and more than 20% is a severe issue [12]. Vitamin A was once thought to have anti-infective properties, and there is mounting evidence that it does affect immune system performance [8]. Numerous studies conducted in regions of the world with usually poor nutritional status have revealed that augmenting preschoolers with vitamin A may condense death and morbidity. According to Humphrey et al. (1996) [13], "An oral dosage of 52 µM given to term children at birth in Indonesia decreased infant death and the frequency of unembellished respirational taints compared to a sample” [8].

Numerous studies conducted in regions of the world with usually poor nutritional status have revealed that complementing infants with vitamin A may reduce mortality and morbidity. According to Humphrey et al. (1996) [13], an oral dosage of 52 µM certain to span of children in Indonesia decreased preschooler death and the frequency of acute respirational distress equated to a sample [14]. In low- and average-money nations, vitamin A deficiency is widespread. According to vitamin A deficiency in young infants, a judicious unembellished public health issue exists in 122 nations; according to recent statistics on worldwide trends in vitamin A insufficiency, rates are much lower in the southeast [15]. In many parts of the world, deaths attributed to vitamin A deficiency have nearly wholly gone, indicating the need to review supplementing practices in light of population demands [15].

Biological effects of vitamin A in infants and preterm infants

Both cellular differentiation and the production of surfactants in the fetal lung depend on vitamin A [16]. The anterior eye needs vitamin A to stay healthy, which is also crucial for the production of the pigment in our eyes [16]. Vitamin A can be used to assess retinal function and can be easily fused into the run. Compact retinal warmth in infants may reflect vitamin A deficiency; apart from the utmost unembellished luggage, electroretinography changes can be reversed with vitamin A supplementation [16]. Symptoms of xerophthalmia, a deficiency affecting older children and adults, are identified [16]. Early gestation is necessary for healthy cardiovascular development, and postnatally, vitamin A speeds up the formation of oxygen-induced ductus arteriosus contraction in the rat model [16,17]. In some studies, retinopathy of prematurity has been linked to low plasma vitamin A concentrations, and retinopathy of prematurity that requires treatment has been related to atypical conjunctival imprint cytology [18]. There is a suggestion that vitamin A supplementation condenses the incidence of air route taint combined. Data indicate a non-significant trend toward a decrease in nosocomial sepsis in newborns receiving vitamin A supplements [16]. Newborns with low hepatic vitamin A levels have a greater commonness and brutality of intraventricular hemorrhage. However, post-delivery vitamin A handling of extremely low birth weight toddlers remained not significantly linked with a decrease in the commonness of heart bleeding [19].

Vitamin A supplementation for infants

One can provide vitamin A intravenously, intramuscularly, or enterally. Vitamin A is administered enterally as a supplement to newborns and kids of all ages since it is well absorbed enterally in term infants (except in malabsorptive conditions). Suppose that sufficiently liberal oral quantities of vitamin A are given to very low birth weight infants, oral vitamin A administration combined with early meals can result in equivalent body fluid concentrations of retinol to intramuscular vitamin A administration [20]. Given that these infants have the utmost possible advantage on or after the add-on of vitamin A, they must be supplemented by a parenteral route, at least in the early stages of life. Even substantial oral amounts of vitamin A from natal do not pointedly raise plasma values of vitamin A or improve outcomes [10,21]. In non-randomized, non-placebo-controlled research, administration with a sole dosage of 200,000 IU of vitamin A (or 100,000 IU if the kid was less than 12 months) was linked to a substantial drop in the lactulose to mannitol ratio (L/M ratio) after two weeks as compared to the baseline level [4]. In non-randomized, non-placebo-controlled research, supplementation with a single dosage of 200,000 IU of vitamin A (or 100,000 IU if the kid was 12 months or older) was linked to a substantial drop in the L/M ratio after two weeks as linked to the reference point level [22]. For use in the field, surveys need one plasma model and may be done with dehydrated plasma samples. The results closely correspond with the disease [23].

**Figure 1:**
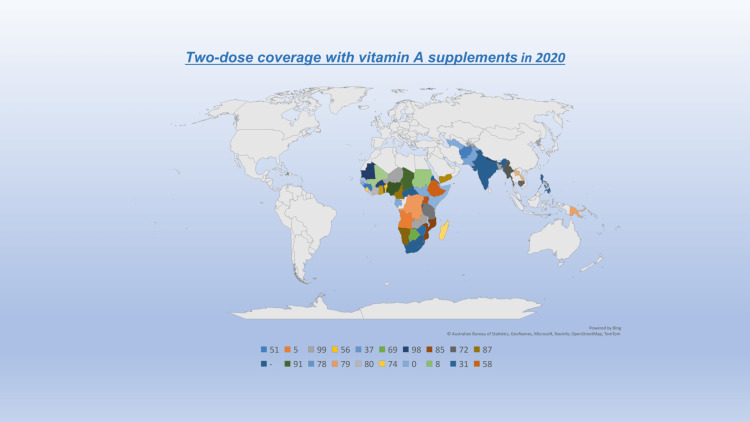
Two-Dose Coverage With Vitamin A Supplements in 2020

It is not advised to administer vitamin A additions to infants between the ages of one and five months as a community well-being measure to lower disease and death. Additional studies would be beneficial to accurate the connotation of vitamin A and its immunological function and to examine regions with significant maternal vitamin A insufficiency. More learning is needed to detect the exemplary markers of vitamin A deficit in this age range [24]. In areas where vitamin A deficiency is a public health issue, it is advised to supplement neonates and children ages six to 59 months with high doses of vitamin A. Improved knowledge of cointerventions that may interact with vitamin A and vaccines is a thing that needs more study [24] (Figure 1). Due to the need for two plasma models in 300 minutes, relative dose response has a low sensitivity but high specificity for vitamin A deficiency [24,25].

Effects of vitamin A supplementation in infants

**Figure 2:**
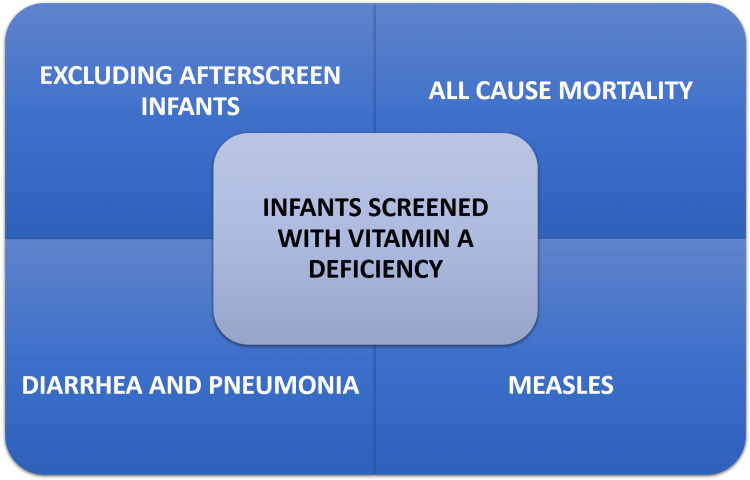
Vitamin A Supplementation and Systematic Identification

There is a tendency toward a decrease in mortality or oxygen usage at one calendar month that is of questionable arithmetical implication when the meta-analysis is limited to the five trials reporting on injection in muscle of vitamin A. When an article by Wardle et al. (2001) [26] is taken into account in the meta-analysis, the result is significantly reduced when babies receiving treatment receive oral vitamin A supplements; the result is significantly reduced [8]. The interpretation of these preliminary study results is challenging due to significant changes in ventilator management and shifting definitions of bronchopulmonary dysplasia; vitamin A administration is often utilized despite the issues these evaluations bring up [27]. Small-scale investigations have raised questions regarding an increased risk of sepsis and necrotizing enterocolitis caused by injection in muscle of vitamin A [27]. Because vitamin A administration to older offspring is frequently administered, the idea that neonatal vitamin A supplementation adversely interacts with future diphtheria-pertussis-tetanus vaccination in females is significant not just for neonates. Many nations advise diphtheria-pertussis-tetanus boosters since many kids are delayed in receiving their first diphtheria-pertussis-tetanus series. Therefore, if vitamin A administration adversely interacts with diphtheria-pertussis-tetanus in females, it could affect older kids [28-31]. Some studies have found that giving older kids vitamin A supplementation and diphtheria-pertussis-tetanus may be hazardous. Regarding the vitamin A supplementation policy for older children, this should cause urgent alarm [7]. Supplementing with vitamin A appears to be an essentially risk-free practice. With the recommended amount of vitamin A administration, a small number of offspring may have modest side effects; nevertheless, they are uncommon and temporary. The most often reported adverse reactions include agitation, wobbly defecation, headaches, temperature, queasiness, and unsettled stomach (in neonates) [32-34]. An indication that vitamin A intake during maternity at the studied levels does not quite appear to lower neonatal or baby mortality may be seen in the pooled estimates for neonatal and infant loss. This is in line with a meta-analysis finding that parental postpartum supplementing did not affect the incidence, while it has not been officially ruled out that neonatal supplementation could have advantages and various trials are now being conducted to investigate this theory. The findings that supplementation had an impact on child malnutrition, notably among the subset of newborns given to evening mothers, support suggestions that this problem is medicated with vitamin A feedstuffs. This is comparable to the results regarding adolescent pregnancy. Although vitamin A is known to influence a child's growth, the precise impact has been challenging to measure since vitamin A insufficiency frequently coexists with certain other growth-restraining factors [35] (Figure 2).

Supplements are affordable and seldom cause adverse side effects. The most efficient dosage and supplementation frequency still need to be studied, although placebo-controlled experiments would be unethical. All at-risk youngsters should continue to receive supplements, especially those in low- and middle-income nations, according to policymakers [36]. To increase child survival and reduce the risk of communicable morbidity, periodic vitamin A supplementation is a helpful public health strategy. Kids with unembellished measles may have an advantage from beginning vitamin A administration due to a short-term rise in antibody production, which may cause an increase in lymphocyte proliferation [4]. Vitamin A's function in healing and maintaining the integrity of the gut mucosa is probably what has an impact on severe diarrhea. The final concluded lower alimentary sufferers and increased nutritional status may also be connected to the apparent advantage for persistence and development in neonates battling human immunodeficiency virus (HIV) [4]. Because vitamin A helps to repair and preserve the integrity of the gut mucosa, it is likely to impact severe diarrhea. It is also possible that the latter, through lowered alimentary sufferers and increased alimentary station, is responsible for the superficial advantage for subsistence and development in neonates battling human immunodeficiency virus (HIV) [37].

Regions within nations that have a high rate of vitamin A deficiency often share dietary and other environmental exposures (e.g., poverty, high levels of infectious diseases, poor development, and inadequate access to food) [38]. Low consumption of vitamin A is frequently attributed to two factors: the high cost and scarcity of foods containing the vitamin in low-income areas. Vulnerable groups have the highest risk of developing vitamin A deficiency and xerophthalmia, according to the World Health Organization (WHO). After weaning young children off breast milk, cereals and starchy roots typically make up most of their diets in low-income areas [22]. By hindering lung repair, increasing squamous cell metaplasia, increasing susceptibility to infection, and reducing the number of alveoli, vitamin A deficiency may induce chronic lung illness. Due to their low vitamin A stress at delivery, newborns with extremely low birth weight are more susceptible to vitamin A insufficiency [19].

## Conclusions

Although their full potential has yet to be reached, supplements have not proven beneficial in helping the poor world achieve its micronutrient demands. The experience with vitamin A supplementation in the latter 1990s was unfavorable. At least one million young children's lives might have been affected due to the widespread usage of these supplements in the 1990s' final years. There are little data that can be used to evaluate the effects of supplement usage on child nutrition, even though infancy is a crucial time for growth and development, and that nutrient shortage, excesses, or imbalances may have significant long-term impacts on health. Supplementing with vitamin A appears to be an essentially risk-free practice. With the recommended amount of vitamin A administration, a small number of offspring may have modest side effects; nevertheless, they are uncommon and temporary. The most often reported adverse reactions include agitation, wobbly defecation, headaches, temperature, queasiness, and unsettled stomach (in neonates). Although vitamin A insufficiency is recognized to affect development, the substantial impact is very challenging to measure since vitamin A deficiency frequently coexists with other growth-restraining defects. Vitamin A supplementation adversely interacts with future diphtheria-pertussis-tetanus vaccination in females. Older kids could also be at risk of vitamin A supplements adversely interacting with diphtheria-pertussis-tetanus. Many nations advise diphtheria-pertussis-tetanus boosters since many kids are delayed in receiving their first diphtheria-pertussis-tetanus series. Vitamin A supplementation has not shown any beneficial effect on the development of term and preterm infants but rather decreases the efficacy of the diphtheria-pertussis-tetanus (DPT) vaccine so the supplementation of vitamin A is rather not advised during infancy.
